# Web services and workflow management for biological resources

**DOI:** 10.1186/1471-2105-6-S4-S24

**Published:** 2005-12-01

**Authors:** Paolo Romano, Domenico Marra, Luciano Milanesi

**Affiliations:** 1 Bioinformatics and Structural Proteomics, National Cancer Research Institute (IST), Genova, I-16132 Italy; 2 Biomedical Technologies Institute (ITB), National Research Council, Segrate (MI), I-20090, Italy; 3 CILEA, Segrate (MI), I-20090, Italy

## Abstract

**Backgorund:**

The completion of the Human Genome Project has resulted in large quantities of biological data which are proving difficult to manage and integrate effectively. There is a need for a system that is able to automate accesses to remote sites and to "understand" the information that it is managing in order to link data properly. Workflow management systems combined with Web Services are promising Information and Communication Technologies (ICT) tools. Some have already been proposed and are being increasingly applied to the biomedical domain, especially as many biology-related Web Services are now becoming available. Information on biological resources and on genomic sequences mutations are two examples of very specialized datasets that are useful for specific research domains.

**Results:**

The architecture of a system that is able to access and execute predefined workflows is presented in this paper. Web Services allowing access to the IARC TP53 Mutation Database and CABRI catalogues of biological resources have been implemented and are available on-line. Example workflows which retrieve data from these Web Services have also been created and are available on-line.

**Conclusion:**

We present a general architecture and some building blocks for the implementation of a system that is able to remotely execute workflows of biomedical interest and show how this approach can effectively produce useful outputs. The further development and implementation of Web Services allowing access to an exhaustive set of biomedical databases and the creation of effective and useful workflows will improve the automation of *in-silico *analysis.

## Background

The Human Genome Project has transformed biology. Since its completion, the field has expanded to the management, processing, analysis and visualization of large quantities of data from genomics, proteomics, medicinal chemistry and drug screening.

This huge amount of data and the heterogeneity of software tools that are used for its distribution make the tasks of searching, retrieving and integrating the information very difficult. Data is typically retrieved and analysed by hand by accessing several bioinformatics servers and by transferring the data by means of FTP clients or web browsers often using the "cut and paste" technique.

Generally speaking, data integration needs stability that in turn can be provided by standardization of information systems, well established domain knowledge, well defined data and well defined goals. Instead, data integration in biology suffers from heterogeneous data and systems, uncertain domain knowledge, fast evolution of data, highly specialized data and the lack of predefined and clear goals.

There is a clear need for a system that can improve information accessibility. Such a system should be able to automate the accesses to the remote sites, in order to retrieve the information from the specific database or to make use of the appropriate software tools to achieve the desired analysis. At the same time, in order to create the correct links between data it should also be able to "understand" the information that it is managing, i.e. its' semantics. Among current Information and Communication Technologies (ICT) technologies, workflow management systems in conjunction with Web Services seems to be the most promising solution.

Workflows are defined by the Workflow Management Coalition as "computerized facilitations or automations of a business process, in whole or part" [1]. Their goal is the implementation of data analysis processes in standardized environments and their main advantages relate to effectiveness, being an automatic procedure they free bio-scientists from repetitive interactions with the web and support good practice, reproducibility over time, reusability of intermediate results and traceability, since the workflow is carried out in a transparent analysis environment where data provenance can be checked and/or controlled.

Some workflow management systems have already been proposed and are being increasingly applied in the biomedical domain. Some of them are add-ons to other tools, like biopipe [2], a perl module designed to be used with bioperl, and GPipe, an extension of the Pise interface [3].

Other systems are autonomous applications that are being developed either by industries, like the Bioinformatic Workflow Builder Interface – BioWBI from IBM [4], and Pipeline Pilot from SciTegic, or by academic and research institutes, like Wildfire from the Singapore Bioinformatics Institute, and Taverna Workbench [5] from the European Bioinformatics Institute (EBI).

Web Services (WS) are software oriented network services which communicate usually by using SOAP (Simple Object Architecture Protocol, a framework for the distribution of XML structured information) over HTTP. They offer a good, standard solution for automated retrieval of information. Standards are available or have been proposed for their retrieval and identification, description and composition [6]. They allow software applications to access data in a semantic- aware way since being in the form of XML documents their contents can be made visible and when metadata is given, interpretation of semantic information becomes possible.

Many WS and WS deployment tools have recently been set up in the biomedical domain and perspectives for their widespread use has been proposed [7,8]. Among the providers of biology oriented WS are some of the most important network service providers such as the USA National Center for Biotechnology Information (NCBI), which has implemented API interfaces for accessing so-called e-utilities [9], the Bioinformatics Center of the National Cancer Institute [10], the Bioinformatics Center of Kyoto University, which has implemented the KEGG API [11], and the European Bioinformatics Institute (EBI).

Biological data are usually found in primary or secondary databases. While the former are the unique reference for all different research domains, the latter are often very specialized databases that are essentially useful for specific domains for which they represent the most important data.

Essential but often underestimated biological information includes biological resources such as microbial strains, human and animal cell lines, plasmids, phages, plant cells and plant cell viruses,. They are collected in specialized centres where they are appropriately characterized and stored with detailed descriptions of the resources being held in catalogues. This information is not only intended to identify single elements of the collection, but also to fully describe their biological properties and special functions. Often catalogues include bibliography references that can help scientists to find further information. The main limitation to accessibility of this information is that catalogues are usually separate and scientists are therefore obliged to search many of them in order to find the resource that is the most appropriate for their research.

Linking information that is available in culture collection catalogues to bioinformatics databanks available in Internet can further significantly improve accessibility of biological resources and can make the collections' holdings more effectively available after a search in molecular biology databanks and vice-versa. As a consequence, additional certified information can be made available to researchers and a growing number of them will hopefully refer to culture collections and make a wider use of biological material of certified quality. In order to promote this, extensions of catalogue information are being carried out at many centres, depending on their research interests.

In oncology, one of the most interesting sources of information relates to mutations, either induced or spontaneous, occurring in genomic sequences and their products. One of the most studied proteins is the human p53 protein (reference sequence is SwissProt P04637) whose importance in the regulation of the apoptosis process in the cell is well known and recognized. This protein can be considered as a tumour suppressor, in the sense that it prevents the occurrence of tumours by promoting apoptosis in pre-cancer, altered cells. Modifications in the regulation of the apoptosis process are positively correlated to the presence of mutations in the TP53 gene (reference sequence is EMBL X02469), thus suggesting that it can, at least partially, be the cause for missing functionality. Moreover, the presence of mutations in the TP53 gene has been observed in about half of all cancers, in the 80% of all colon cancer tumours, in the 50% of lung cancer tumours, and in the 40% of breast cancer tumours.

We present here the architecture of a system that has been designed to manage, organize and execute a set of predefined and tested workflows of interest to oncology. We also present a set of Web Services which allows the researcher to search and retrieve information from CABRI catalogues of biological resources and our implementation of the IARC TP53 Mutation Database. Finally, we present some of the workflows that have been designed with the twofold aim of allowing access to above Web Services and of representing an example of data integration that can be achieved through workflow management systems.

## Methods

### The system architecture

**Figure 1:**
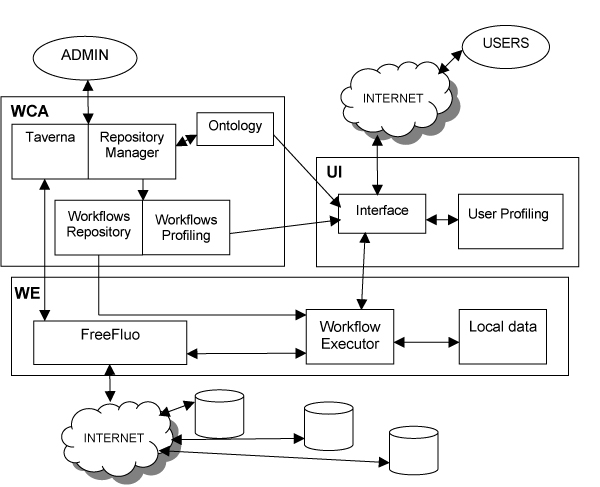
**General architecture of the system. **The general architecture of the system includes three blocks: workflows creation and annotation (WCA), user interface (UI) and workflows execution (WE). The "Taverna" and "FreeFluo" boxes refer to open source software developed externally from the project. The boxes named "Repository Manager", "Workflow Executor" and "Interface" refer to purpose applications developed within the project. All other boxes refer to databases, either local or remote.

We have designed a general architecture of a system (see figure 1) for the management, organization and execution of workflows of biomedical interest that are intended to access to and to retrieve data from various Web Services.

The system is partially based on open source software tools. The Taverna Workbench is a workflow management system developed at the European Bioinformatics Institute (EBI) as part of the ^my^Grid project [23]. It allows skilled end users to create complex analysis workflows, to access both remote and local processors of various kinds, to run workflows and to display results in different formats. In Taverna workflow execution is carried out by an associated tool, FreeFluo. Taverna also includes an ontology for bioinformatics data. Its only requirement is the availability of Java Run-time Environment (JRE) on a Windows XP or Linux box. A MySQL data management system [12] can optionally be used for local data storage.

The system includes three main blocks: the workflow manager, the user interface and the workflow executor. Workflows are created and tested by an administrator using the Taverna Workbench. They are then stored in a repository by adopting the Taverna Simple conceptual unified flow language (Scufl) format. At the same time, their main processing steps are annotated in workflows profiles by using a specially designed ontology. This ontology describes bioinformatics tasks on the basis of their input and output data, processing type and application domain. These main processing steps may actually represent more real tasks grouped to achieve a functionally significant processing step.

The user interface supports end users authentication and profiling and allows for the selection and launch of workflows. Workflow selection can be assisted by the user profile and by searching through annotated workflows significant processors.

Workflows are executed by the third block that is based on FreeFluo and it is also able to store input and output data of actual workflow executions, so that they can later be analysed and possibly reused.

### The implementation of CABRI databases

Common Access to Biological Resources and Information (CABRI) [13] is a network service for the distribution of resources and related data that are collected and managed by a number of European culture collections. Through the CABRI service, more than 110,000 biological resources from 28 collections can be searched in a single site and by means of a single query. Searching abilities include queries by scientific name and by strain number and a free text search. Search by scientific name includes an option for adding synonyms' support. Through CABRI services, resources can also be pre-ordered on-line by using an electronic shopping cart.

Links from CABRI catalogues to other databanks available on-line and vice-versa were added as part of the the European Biological Resource Centres Network (EBRCN) project [14], funded by the EU from 2001 to 2004. This effort included links to and from catalogues and the EMBL Data Library, Medline and plasmids maps (see table 1).

CABRI catalogues have also been implemented in SRS (Sequence Retrieval System), a well known search engine for biomedical databanks [15]. This was done because SRS has a number of useful features: it is able to manage heterogeneous database formats, including flat files, relational databases and XML files; it has a simple and effective interface, it provides both internal inter-database links and HTML links to external information sources, it offers a specific link operator that allows for powerful search and display options, new databases can easily be added and data indexes can be created in a very flexible way.

The implementation was carried out by first comparing the data structures and contents of all collection databases and then defining common data sets, unique for the different kind of resources. Guidelines for data input and authentication have also been defined and agreed upon. These often include references to common data sets and vocabularies [16].

For each catalogue, information is completed with reference data sets referring, e.g., to culture media, synonyms, hazard and restrictions to distribution. For many catalogues, explicit links have been defined to Medline and plasmids' maps, while links from EMBL Data Library to CABRI catalogues have been added in the EMBL version 81.

### The implementation of the IARC TP53 Mutation Database in SRS

The TP53 Mutation Database [17] of the International Agency for the Research on Cancer (IARC) currently is the biggest and most detailed among databases collecting information on TP53 mutations described in the literature. It includes somatic mutations (mutation type with references, mutation prevalence, mutation and prognosis), germline mutations (both data and references), polymorphisms, mutant functions and cell line status.

Release 10 includes 21,587 somatic mutations whose description has been derived from 1,876 papers, 1,839 of which are included in the Medline database. Information on somatic mutations includes data on the mutation, the sample, the patient and his/her lifestyle. Reference vocabularies and standardized annotations are used extensively for the description of the mutation, tumour site, type and origin and for literature references. Examples of the former are ICD-O (International Classification of Diseases – Oncology) and SMOMED nomenclatures.

This database is available through the IARC web site [18], where queries can only be executed on-line and imply human interaction. Moreover, there are datasets (e.g., mutation and prognosis, germline mutations, polymorphisms and cell lines status) that are not searchable on-line. Further accessibility limitations include the lack of links to other bioinformatics databases and tools and the user interface, which is based on an interactive query form therefore making automated data retrieval difficult.

The IARC TP53 Mutation Database and all its data subsets have been downloaded and reformatted for easier indexing and searching through SRS. Reformatting involved a few changes and additions in the database fields and their contents. All datasets have then been implemented as separate libraries. Data fields have been defined in agreement with controlled vocabularies that were used for data input at IARC, so that SRS indexes are based on them as well.

All IARC TP53 Database datasets have been made available on-line and can easily and effectively be queried through SRS query forms [19]. Due to the careful definition of data fields, terms included in the controlled vocabularies that were used during data input at IARC can also be used from within the SRS extended query form, thus allowing for a data-driven search.

### Implementation of Web Services and workflows

Some original Web Services have been implemented. They allow the retrieval of information from CABRI and TP53 databases through a programatic interface, by also allowing for the inclusion of calls in pipelines and workflows management systems. We implemented Web Services allowing for the execution of a search either by name, by identifier or free text to CABRI catalogues and of a search by interesting properties towards the IARC TP53 Mutation Database.

Integration of these data with other sources was planned by using either unique IDs or common terms. In order to do this, two types of services were implemented, searching either for a specific feature and returning IDs or searching for an ID and returning full records.

Web Services were implemented by using Soaplab, a SOAP-based Analysis Web Service providing a programatic access to applications on or through remote computers [20,21]. Soaplab allows for the implementation of Web Services offering access to local command-line applications, like the EMBOSS software, and to the contents of ordinary web pages. The only requirements of Soaplab are the Apache Tomcat servlet engine with the Axis SOAP toolkit, Java and, optionally, perl and mySQL.

**Figure 2:**
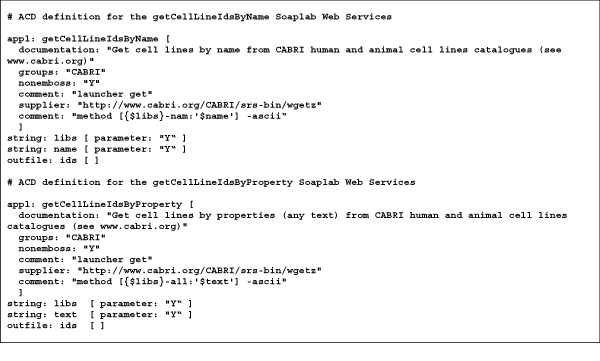
**ACD definitions for two Soaplab based Web Services searching CABRI cell lines catalogues. **This figure shows the definitions used for the set-up of the Web Services that return unique identifiers of cell lines in CABRI catalogues after a search either by name or by property (free text search). These definitions are written in the ACD language and must be converted into XML before they can be used. Differences between the two definitions consist in the comment line where the actual parameters for the remote call to the CABRI site are defined

Once the server has been installed new Web Services are added by defining simple definitions of the related execution commands (see figure 2) written in the ACD language which are then converted into XML before they can be used. In our case, all Web Services have been defined as remote calls to SRS sites. Essential differences between Web Services definitions consist in the input and output parameters and in the specification of the URL address where these parameters must be inserted.

## Results

A set of Web Services have been designed and implemented by using Soaplab and are now available on-line, both through their own WSDL descriptions [24] and the Soaplab server. They implement access to IARC TP53 Mutation Database and to CABRI catalogues of biological resources. Lists of currently available Web Services are presented in tables 2 and 3.

**Figure 3:**
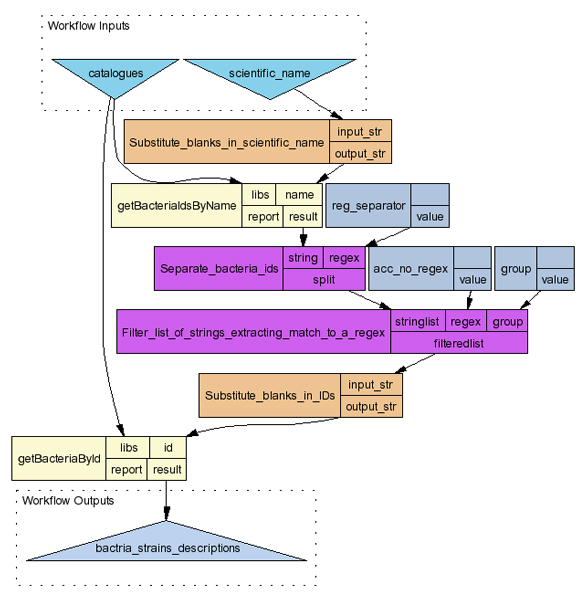
**Diagram of the GetBacteriaByName workflow. **This diagram lists one of the workflows that have been created and are available on-line for demonstration purposes. Two web services are used (getBacteriaIdsByname and getBacteriaById), together with some local string elaborations. This output has been generated by the Taverna Workbench.

Workflows have been designed to access to and to retrieve data from the CABRI and IARC TP53 Mutations Database Web Services. They can also be viewed as an example of data integration that can be achieved through workflow management systems. Scufl formatted versions of these workflows have been created and are available on-line [22] for use by interested users working with Taverna Workbench. In table 4, some workflows are briefly presented, while in figure 3 the diagram of the GetBacteriaByName workflow is shown and described.

## Conclusion

We have presented in this paper a general architecture for the implementation of a system that is able to execute workflows of biomedical interest remotely. We have presented as well some Web Services and workflows that have been designed and implemented to allow automated retrieval through a programatic interface of information related to CABRI catalogues of biological resources and to the IARC TP53 Mutations Database.

The further development and implementation of Web Services allowing the access to and retrieval from an exhaustive set of molecular biology and biomedical databases being carried out by many research centres and network service providers in the biological and medical domains and the creation of effective and useful workflows by interested scientists through widely distributed workflows management systems such as those presented in this paper will significantly improve automation of *in-silico *analysis.

## Figures and Tables

**Table 1:** External links to/from CABRI catalogues.

**Catalogue**	**EMBL Links**	**Medline Links**	**Map Links**
ICLC Human and animal cell lines	0	294	0
DSMZ Human and animal cell lines	0	905	0
NCCB Plasmids	0	371	0
BCCM/LMBP™ Plasmids	154	405	816
NCCB Phages	0	30	0
BCCM/LMG™ Bacteria strains	799	0	0
DSMZ Bacteria strains	2,618	0	0
NCIMB Bacteria strains	334	0	0
CIP Bacteria strains	545	0	0
BCCM/IHEM™ Fungi and Yeasts strains	18	0	0
BCCM/MUCL™ Fungi and Yeasts strains	119	0	0
CBS Filamentous fungi strains	9,667	454	0
CBS Yeasts strains	52,983	1,132	0
CABI Bioscience Fungi strains	206	0	0
DSMZ Fungi and Yeasts strains	31	0	0
Total	67,256	3,591	816

The reported figures represents the number of external links to/from CABRI catalogues. Only catalogues having at least one link are listed. EMBL links are included in the EMBL Data Library since EMBL version 81. Links to Medline and plasmids' maps are included in CABRI catalogues' records.

**Table 2:** List of Web Services related to IARC TP53 Mutation Database.

**Web Service Name**	**Input**	**Output**
getP53MutationsByProperty	lib, text	Full record
getP53MutationsByIds	Id	Full record
getP53MutationIdsByType	lib, mutation type	id(s)
getP53MutationIdsByEffect	lib, effect	id(s)
getP53MutationIdsByExon	lib, exon number	id(s)
getP53MutationIdsByIntron	lib, intron number	id(s)
getP53MutationIdsByCodonNumber	lib, codon number	id(s)
getP53MutationIdsByCpgSite	lib, cpg site (true/false)	id(s)
getP53MutationIdsBySpliceSite	lib splice site (true/false)	id(s)
getP53MutationIdsByMetastasisLocalization	lib, metastasis localization (organ)	id(s)
getP53MutationIdsByTumorOrigin	lib, origin (primary, secondary, ...)	id(s)

This table lists all Web Services that have been created with the aim of allowing data retrieval from IARC TP53 Mutation Database. Here id refers to the Mutation_ID defined by IARC, while lib refers to the name of involved database that currently is 'tp53_iarc' but has been parameterized in view of future extension of the system, allowing the search of other databases as well.

**Table 3:** List of Web Services related to CABRI catalogues.

**Web Service Name**	**Involved catalogues**	**Input**	**Output**
getBacteriaIdsByName	Bacteria strains	lib(s), name	id(s)
getBacteriaIdsByProperty	Bacteria strains	lib(s), text	id(s)
getBacteriaById	Bacteria strains	id	full record
getFungiIdsByName	Filamentous fungi strains	lib(s), name	id(s)
getFungiIdsByProperty	Filamentous fungi strains	lib(s), text	id(s)
getFungiById	Filamentous fungi strains	id	full record
getYeastIdsByName	Yeasts strains	lib(s), name	id(s)
getYeastIdsByProperty	Yeasts strains	lib(s), text	id(s)
getYeastsById	Yeasts strains	id	full record
getPlasmidIdsByName	Plasmids	lib(s), name	id(s)
getPlasmidIdsByProperty	Plasmids	lib(s), text	id(s)
getPlasmidsById	Plasmids	id	full record
getPhageIdsByName	Phages	lib(s), name	id(s)
getPhageIdsByProperty	Phages	lib(s), text	id(s)
getPhagesById	Phages	id	full record
getCellLinesIdsByName	Human and animal cell lines	lib(s), name	id(s)
getCellLinesIdsByProperty	Human and animal cell lines	lib(s), text	id(s)
getCellLinesById	Human and animal cell lines	id	full record
getResourceIdsByName	All	lib(s), name	id(s)
getResourcesById	All	id	full record

This table lists all Web Services that have been created with the aim of allowing data retrieval from CABRI catalogues. Here, id refers to the unique identifier of resources in the catalogues and can therefore be a strain number, an accession number or a collection number. Instead, lib refers to the name of involved catalogues, while name is the scientific name where spaces must be substituted by the '_SP_' string and text is any single word.

**Table 4:** List of some workflows. .

**Workflow name and description**	**Inputs**	**Outputs**
**GetMutationsByIntron** Retrieves all mutations occurring in the specified intron	**lib**: database name ('tp53_iarc') **intron**: intron where mutation occurs (integer)	**ids**: list of mutations' ids **mutations**: full records
**GetTP53MutationsByIntronAndEffect** Retrieves all TP53 mutations occurring in the specified intron and having the specified effect	**intron**: intron where mutation occurs (integer) **effect**: mutation effect (fs, missense, na, nonsense, other, silent, splice)	**ids**: list of mutations' ids **mutations**: full records
**GetTP53MutationsByIntronAndEffect2** Retrieves all TP53 mutations that: • occurs in the specified intron OR have the specified effect • occurs in the specified intron AND have the specified effect • occurs in the specified intron BUT DOES NOT have the specified effect	**intron**: intron where mutation occurs (integer) **effect**: mutation effect (fs, missense, na, nonsense, other, silent, splice)	**mutation_in_or**: full records of mutations retrieved by applying a logical OR to results obtained by single queries **mutation_in_but_not**: full records of mutations retrieved by removing results of the second query from results of the first query **mutation_in_and**: full records of mutations retrieved by applying a logical AND to results obtained by single queries
**GetBacteriaByName** Retrieves full descriptions of bacteria strains from specified CABRI catalogues, given the scientific name	**catalogues**: name(s) of CABRI bacteria catalogues **scientific name**: genus and species of the desired strains	**bacteria_strains_description**: full records of selected bacteria strains

This table lists some of the workflows that have been created and are available on-line for demonstration purposes in the Scufl format. These workflows can be launched by using the Taverna Workbench

## References

[B1] Workflow Management Coalition (WfMC). http://www.wfmc.org/.

[B2] Hoon S, Kumar Ratnapu J, Chia J, Kumarasamy B, Juguang X, Clamp M, Stabenau A, Potter S, Clarke L, Stupka E (2003). Biopipe: A Flexible Framework for Protocol-Based Bioinformatics Analysis. Genome Research.

[B3] Letondal C (2001). A Web interface generator for molecular biology programs in Unix. Bioinformatics.

[B4] Life Sciences Practice Team (2004). BioWBI and WEE: Tools for Bioinformatics Analysis Workflows.

[B5] Oinn T, Addis M, Ferris J, Marvin D, Senger M, Greenwood M, Carver T, Glover K, Pocock MR, Wipat A, Li P (2004). Taverna: a tool for the composition and enactment of bioinformatics workflows. Bioinformatics.

[B6] Web Services activities at the World-Wide Web Consortium (W3C). http://w3.org/2002/ws/.

[B7] Stein L (2002). Creating a bioinformatics nation. Nature.

[B8] Jamison DC (2003). Open Bioinformatics. (editorial) Bioinformatics.

[B9] Entrez Utilities Web Service at NCBI. http://eutils.ncbi.nlm.nih.gov/entrez/query/static/esoap_help.html.

[B10] Web Services at NCICB. http://ncicb.nci.nih.gov/.

[B11] KEGG Web Services. http://www.genome.jp/kegg/soap/.

[B12] MySQL data management system. http://www.mysql.com/.

[B13] Common Access to Biological Resources and Information (CABRI) network service. http://www.cabri.org/.

[B14] European Biological Resource Centres Network (EBRCN). http://www.ebrcn.org/.

[B15] Etzold T, Ulyanov A, Argos P (1996). SRS: information retrieval system for molecular biology data banks. Meth Enzymol.

[B16] CABRI Guidelines for Catalogue Production: data input and authentication. http://www.cabri.org/guidelines/catalogue/CPdata.html.

[B17] Olivier M (2002). The IARC TP53 Database: new online mutation analysis and recommendations to users. Hum Mutat.

[B18] TP53 Mutation Database at IARC. http://www-p53.iarc.fr/.

[B19] SRS implementation of IARC TP53 Mutation Database. http://srs.o2i.it/.

[B20] Soaplab. http://www.ebi.ac.uk/soaplab/.

[B21] Senger M, Rice P, Oinn T (2003). Soaplab – a unified Sesame door to analysis tools. Proceedings, UK e-Science, All Hands Meeting Editors – Simon J Cox.

[B22] Demo Workflows. http://www.o2i.it/workflows/.

[B23] Stevens R, Robinson A, Goble C (2003). ^my^Grid: personalised bioinformatics on the information grid. Bioinformatics.

[B24] Oncology over Internet Soaplab server. http://www.o2i.it:8080/axis/services.

